# Overexpressing high levels of human vaspin limits high fat diet-induced obesity and enhances energy expenditure in a transgenic mouse

**DOI:** 10.3389/fendo.2023.1146454

**Published:** 2023-04-19

**Authors:** Inka Rapöhn, Ivet Elias, Juliane Weiner, Anna Pujol, Stephanie Kehr, Alexandra Chadt, Hadi Al-Hasani, Ralph Burkhardt, Nora Klöting, Michael Stumvoll, Fatima Bosch, Peter Kovacs, John T. Heiker, Jana Breitfeld

**Affiliations:** ^1^ Helmholtz Institute for Metabolic, Obesity and Vascular Research (HI-MAG) of the Helmholtz Zentrum München at the University of Leipzig and University Hospital Leipzig, Leipzig, Germany; ^2^ Center of Animal Biotechnology and Gene Therapy (CBATEG) and Department of Biochemistry and Molecular Biology, Universitat Autònoma de Barcelona, Bellaterra, Spain; ^3^ Centro de Investigación Biomédica en Red de Diabetes y Enfermedades Metabólicas Asociadas (CIBERDEM), Madrid, Spain; ^4^ Medical Department III - Endocrinology, Nephrology, Rheumatology, University of Leipzig Medical Center, Leipzig, Germany; ^5^ Bioinformatics Group, Department of Computer Science and Interdisciplinary Center for Bioinformatics, Leipzig University, Leipzig, Germany; ^6^ Institute for Clinical Biochemistry and Pathobiochemistry, German Diabetes Center (DDZ), Leibniz Center for Diabetes Research at Heinrich Heine University Düsseldorf, Düsseldorf, Germany; ^7^ German Center for Diabetes Research (DZD), München-Neuherberg, Germany; ^8^ Institute of Clinical Chemistry and Laboratory Medicine, Transfusion Medicine, University Hospital Regensburg, Regensburg, Germany

**Keywords:** serpin, metabolic disease, obesity, adipose tissue, mouse model, inflammation

## Abstract

Adipose tissue inflammation and insulin resistance are hallmarks in the development of metabolic diseases resulting from overweight and obesity, such as type 2 diabetes and non-alcoholic fatty liver disease. In obesity, adipocytes predominantly secrete proinflammatory adipokines that further promote adipose tissue dysfunction with negative effects on local and systemic insulin sensitivity. Expression of the serpin vaspin (SERPINA12) is also increased in obesity and type 2 diabetes, but exhibits compensatory roles in inflammation and insulin resistance. This has in part been demonstrated using vaspin-transgenic mice. We here report a new mouse line (h-vaspinTG) with transgenic expression of human vaspin in adipose tissue that reaches vaspin concentrations three orders of magnitude higher than wild type controls (>200 ng/ml). Phenotyping under chow and high-fat diet conditions included glucose-tolerance tests, measurements of energy expenditure and circulating parameters, adipose tissue and liver histology. Also, *ex vivo* glucose uptake in isolated adipocytes and skeletal muscle was analyzed in h-vaspinTG and littermate controls. The results confirmed previous findings, revealing a strong reduction in diet-induced weight gain, fat mass, hyperinsulinemia, -glycemia and -cholesterolemia as well as fatty liver. Insulin sensitivity in adipose tissue and muscle was not altered. The h-vaspinTG mice showed increased energy expenditure under high fat diet conditions, that may explain reduced weight gain and overall metabolic improvements. In conclusion, this novel human vaspin-transgenic mouse line will be a valuable research tool to delineate whole-body, tissue- and cell-specific effects of vaspin in health and disease.

## Introduction

Obesity is the major risk factor for type 2 diabetes (T2D), atherosclerosis and nonalcoholic fatty liver disease (1). Inflammation of the adipose tissue (AT) is a frequent consequence of adipocyte hypertrophy, increased AT stresses and immune cell infiltration, and contributes to the development of insulin resistance (2, 3).

Visceral adipose tissue derived serine protease inhibitor (vaspin), also known as SERPINA12, is a member of the serpin family (4), that was first identified in visceral (omental) AT of the Otsuka Long-Evans Tokushima fatty (OLETF) rat, an animal model of obesity and T2D (5). In humans, serum vaspin levels are increased in individuals with obesity and T2D (6). Treatment of obese and insulin resistant mice with recombinant vaspin counteracts obesity-induced inflammation and dysfunction of adipose tissue and liver and partly restores glucose tolerance (7–9). In line, metabolic health of mice overexpressing vaspin is more resistant to obesogenic conditions while a vaspin knockout aggravates metabolic dysfunction in obesity (10). Together, these findings have established the general view of vaspin as a beneficial and compensatory player in obesity-related disorders and diseases (11, 12).

The serpin vaspin inhibits two kallikrein-related peptidases, kallikrein 7 (KLK7) and 14 (KLK14) (8, 13). Knockout of KLK7 in AT dampened obesity-induced AT inflammation and preserved insulin sensitivity in mice (14). Also, a whole-body knockout of KLK7 improved diet-induced metabolic alterations with more pronounced reduction of diet-induced weight gain (15). Anti-inflammatory effects of vaspin in the liver have been in part ascribed to vaspins’ interaction with the ER chaperon GRP78 on the cell surface of hepatocytes (10).

The protective effects of vaspin in obesity-induced metabolic dysfunctions induced by obesity have been demonstrated using a transgenic mouse model (m-vaspinTG) overexpressing mouse vaspin in AT (10). This model exhibited an increase of circulating vaspin levels by 3-fold (0.6 ng/ml in vaspin transgenic mice vs 0.2 ng/ml in wildtype controls). A mouse model to specifically assess the function of human vaspin in metabolic disease has not been reported. We here report a second mouse line (h-vaspinTG) with transgenic expression of human vaspin under the *aP2* promotor that reaches vaspin concentrations three orders of magnitude higher than wild type controls (>200 ng/ml). In line with previous findings, phenotypic characterization of the h-vaspinTG mice revealed a strong reduction in diet-induced weight gain, fat mass, hyperinsulinemia, -glycemia and -cholesterolemia. This mouse represents an ideal model for future investigations of vaspin function in obesity-related diseases, such as atherosclerosis and T2D.

## Materials and methods

### Generation of vaspin-transgenic mice (h-vaspinTG)

A pET16b plasmid containing the human *SERPINA12* sequence (excluding the signal sequence and cloned using *NdeI* and *BamHI*) was provided by Prof. Jun Wada (Okoyama University Graduate School of Medicine) and served as basis for the construction of further plasmids. First, the N-terminal sequence of the signal peptide was added by PCR and the whole construct was cloned into the *EcoRI* and *XhoI* sites of the pcDNA 3.1(-) expression plasmid. The correctness of the sequence was checked by sequencing. In the following steps, the whole *SERPINA12* gene was cloned into the *SmaI* site of a plasmid containing the aP2-promotor and a SV40polyA to achieve a vaspin overexpression in the AT. Finally, transgenic mice expressing the *aP2- SERPINA12* chimeric gene were obtained by microinjection of oocytes from B6SJL (F2) mice, which was conducted in the Unitat d´Animals Transgènics (UAT-CBATEG) of the Universitat Autònoma de Barcelona, Bellaterra, Spain. Twelve founders (1 male, 11 females) were obtained and Southern blot analysis was performed using *EcoRI* digested genomic DNA. Serum vaspin concentrations were measured by ELISA (Adipogen, Korea). Further genotyping of offspring mice was done by PCR using human specific *SERPINA12* sequence primers, available upon request. Resulting h-vaspinTG mouse lines were then backcrossed on the C57Bl/6N background for 5 generations before phenotyping.

### Animals

Mice were housed under pathogen-free conditions (3-5 mice per cage) at 23°C on a 12 h light/dark cycle in the local animal facility (MEZ - Medizinisch-Experimentelles Zentrum, University of Leipzig, Leipzig). All mice had *ad libitum* access to water and food and were either fed a standard chow diet (V1534, 9 kJ% from fat, Ssniff^®^, Soest, Germany) or kept on a high fat diet (HFD; containing 59 kJ% from fat; E15772-34, Ssniff^®^, Soest, Germany) starting at an age of six weeks for up to 30 weeks. Non-transgenic littermates served as controls. After sacrificing, serum was taken and organs were removed, weighed (only liver, subcutaneous inguinal (iWAT), visceral epididymal (eWAT), brown adipose tissue (BAT)), snap-frozen in liquid nitrogen and stored at -80°C until further use. All animal experiments were approved by the local authorities (for Leipzig: Landesdirektion Leipzig, State of Saxony, Germany, reference number TVV26-16; for Düsseldorf: Ethics Committee of the State Ministry of Agriculture, Nutrition and Forestry, State of North Rhine-Westphalia, Germany, reference number 84-02.04.2016.A430; for Barcelona: Ethics Committee in Animal and Human Experimentation of the Universitat Autònoma de Barcelona, Spain), as recommended by the responsible local animal ethics review boards.

### Phenotypic characterization of h-vaspinTG mice

Male mice were studied from the age of six up to 29 weeks on either chow or HFD. Body weight was recorded weekly. Intraperitoneal (i.p.) glucose tolerance tests (GTT; at 25 weeks of age, 2 g glucose per kg body weight), body composition analysis and indirect calorimetry (at 28 weeks of age, for 72 h) were performed as previously described (14). GTTs were performed after an overnight fast. GTT area under the curve (AUC) was calculated for each individual mouse after subtraction of the basal glucose level. Blood glucose was determined from whole-venous blood samples using an automated glucose monitor (FreeStyle Mini; Abbott GmbH, Ludwigshafen, Germany).

Serum adiponectin, leptin, insulin, C-peptide and vaspin were measured by standard ELISA; serum HbA1c, triglycerides (TG) and cholesterol (chol) were analyzed by an automated chemical analyzer at the Institute of Laboratory Medicine and Clinical Chemistry, Medical Department, University of Leipzig (14). AT and liver histology and measurements of adipocyte size distribution was performed as described previously (14).

### Gene expression analysis

Total RNA was isolated from various mouse tissues using TRIzol (Life Technologies, Grand Island, NY). RNA clean-up was done using RNeasy MinElute Cleanup (Qiagen) according to the manufacturer’s instructions. Following, 500 ng RNA was reverse transcribed with standard reagents (Life Technologies). Human and mouse gene expression was measured by quantitative real-time q-PCR using TaqMan methodology and fluorescence was detected on an ABI PRISM 7500 sequence detector (Applied Biosystems, Darmstadt, Germany). The following assays were used: Hs00545180_m1 for the human *SERPINA12* gene; Mm00471557_m1 for endogenous mouse *serpinA12* gene. Adipocyte gene expression was calculated by ΔΔCT method and normalized to *36b4 l*evels in each sample.

### *Ex vivo* glucose uptake into isolated skeletal muscle and mature adipocytes

Glucose uptake was assessed in intact isolated Extensor digitorum longus (EDL) skeletal muscles by the accumulation of [^3^H]2-deoxyglucose (Hartmann Analytic; Braunschweig, Germany) and using [^14^C]mannitol (PerkinElmer, Waltham, MA) as an extracellular marker as described (16). Briefly, mice were sacrificed and EDL muscles were removed. After recovery, muscles were treated with or without 120 nM insulin (Actrapid, Novo Nordisk) for 30 min. Finally, muscles were treated with 1 mM [^3^H]2-deoxy-glucose (2.5 mCi/mL) and 19 mM [^14^C]mannitol (0.7 mCi/mL) for 20 minutes, subsequently frozen in liquid nitrogen. Cleared protein lysates were used to determine incorporated radioactivity by scintillation counting.

Glucose uptake into isolated white adipocytes was assessed *ex vivo* by measuring the incorporation of radioactively labelled [^14^C]-D-glucose (Hartmann Analytic, Braunschweig, Germany) as described (17). Briefly, epididymal fat pads were dissected, primary adipocytes were isolated by collagenase digestion and treated with or without 120 nM insulin for 30 min, followed 0.1 µCi/µl [^14^C]-D-glucose for 30 min. Cells were centrifuged in tubes containing dinonyl phthalate oil and the incorporated radioactivity was determined by scintillation counting. The resulting counts were normalized to the lipid weight of the samples using overnight heptane extraction.

### Statistical analysis

Data are presented as means ± SEM. Statistical analyses were performed using GraphPad Prism 9 (GraphPad, San Diego, CA, USA), except where otherwise indicated. Methods of statistical analyses were chosen according to the design of each experiment and are indicated in the figure legends. If not otherwise stated, an adjusted p < 0.05 was considered statistically significant.

## Results

### Vaspin transgenic mice present highly supraphysiological levels of circulating vaspin

**Figure 1:**
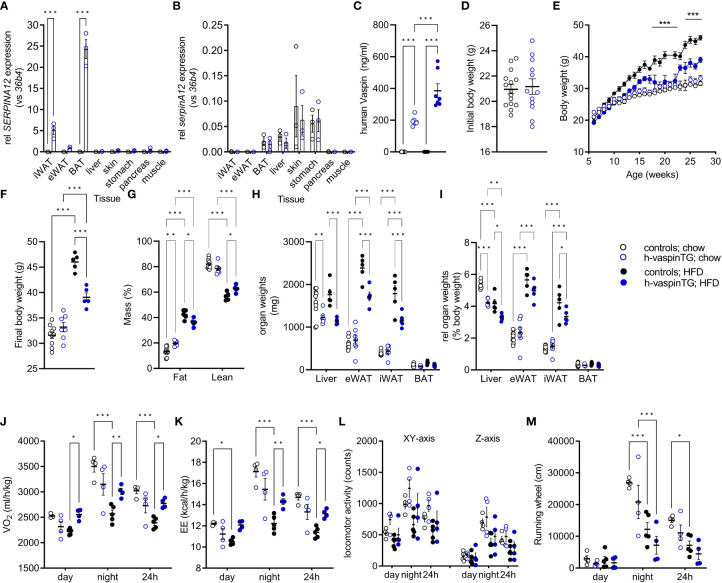
Characterization of h-vaspinTG mice and controls under chow and high fat diet (HFD). **(A, B)** relative mRNA expression of **(A)** human (*SERPINA12*) and **(B)** endogenous (*SerpinA12*) vaspin in AT depots and peripheral tissues as determined by RT-qPCR (n = 3 per group). Gene expression was normalized to *36b4*. **(C)** Human vaspin serum concentration (ng/ml) as measured by ELISA (n = 7-10), at the end of the study. **(D)** Initial body weight, **(E)** body weight gain and **(F)** final body weight of h-vaspinTG and control mice fed a chow or a HFD for 29 weeks, starting at the age of 6 weeks (n = 5-10). **(G)** Percentage of fat mass (%) and lean body mass (%) as determined by EchoMRI at the end of the study (n = 5-10). **(H, I)** Absolute and relative organ weights of liver, epididymal (eWAT), inguinal (iWAT) and brown adipose tissue (BAT) at the end of the study (n =5-10). **(J-M)** Indirect calorimetry of h-vaspinTG and control mice under chow and HFD during light and dark phase (n = 4-5 per group). **(J)** Oxygen consumption (VO_2_ in ml/kg/h) and **(K)** energy expenditure (EE in kcal/h/kg) were recorded over a period of 72 h. **(L)** Physical activity assessed *via* locomotor activity as movement in cage (XY-axis), rearing (Z-axis) and **(M)** running in wheel (cm) were recorded for 72 h (n = 4-5). Data are presented as mean ± SEM. Statistical significance was calculated by two-way ANOVA followed by Šidák’s **(A, B, D)** or Tukey’s **(G-L)** *post-hoc* test or by one-way ANOVA with Fisher’s LSD test **(C)** (*p ≤ 0.05, ** p ≤ 0.01, ***p ≤ 0.001).

As reported above, 12 founders (1 male and 11 females) were obtained after microinjection of oocytes. Quantification of AT mRNA expression in these h-vaspinTG mice revealed a high-level expressing mouse, five intermediate expressing and six low expressing founders (data not shown). For further analysis, male mice of the high-level vaspin expressing line were phenotyped in detail. Expression of human vaspin (*SERPINA12*) was only detectable in all AT depots and highest in BAT, followed by iWAT and rather low in eWAT (**Figure 1A**). Non-transgenic controls were negative for *SERPINA12* mRNA expression in all tissues. Gene expression of endogenous vaspin (*serpinA12*) was not different between h-vaspinTG and controls, with highest expression in skin, stomach, liver and BAT (**Figure 1B**). A previously reported vaspin transgenic mouse line exhibited a 2-3-fold increase (0.4-0.6 vs. 0.2 ng/ml) in endogenous (mouse) circulating vaspin levels compared to controls (10). Here, our high-level expressing h-vaspinTG mice exhibited similar expression levels of serum endogenous vaspin (data not shown) and high circulating levels of human vaspin (200 ng/ml; **Figure 1C**). Non-transgenic controls were negative for human vaspin in the serum. H-vaspinTG mice fed a HFD for 29 weeks showed an additional significant increase to ~400 ng/ml of circulating vaspin (**Figure 1C**), demonstrating highly supraphysiological levels of vaspin in our h-vaspinTG mice that correspond to a 4.000 – 8.000-fold increase compared to controls.

### Vaspin transgenic mice are less sensitive to diet-induced obesity

At the beginning of the study, h-vaspinTG and controls had comparable body weights (**Figure 1D**) and body length (**Table 1**). Both lines responded to HFD feeding, gaining significant body weight compared to their chow-fed controls (**Figure 1E**). After 29 weeks on the respective diets, h-vaspinTG and controls did not significantly differ in body weight under chow diet conditions, though h-vaspinTG were heavier (33.2 ± 0.9 g vs. 31.6 ± 0.6 g; **Figure 1F**). When fed a HFD, h-vaspinTG responded much less to the diet with significantly lower body weights after 10 weeks on the diet. At the end of the study, h-vaspinTG had gained ~5.5 g less body weight compared to controls (39.1 ± 0.9 g vs. 46.0 ± 0.7 g; **Figures 1E, F**).

**Table 1:** Anthropometric and metabolic parameters in male chow and high-fat diet (HFD)-fed h-vaspinTG and control mice.

	Chow		HFD	
	Control (n)	h-vaspinTG (n)	Control (n)	h-vaspinTG (n)
Body length (cm)	9.7 ± 0.1 (10)	9.6 ± 0.1 (7)	10.0 ± 0.1 (5)	9.8 ± 0.1 (5)
Tail length (cm)	8.2 ± 0.1 (10)	8.3 ± 0.1 (7)	8.0 ± 0.1 (5)	7.8 ± 0.2 (5)
Body temp. (°C)	35.2 ± 0.6 (7)	35.2 ± 0.4 (7)	35.4 ± 0.6 (5)	35.3 ± 0.5 (5)
Food intake (kcal/d)	20.6 ± 1.6 (4)	21.3 ± 4.8 (4)	20.7 ± 2.7 (5)	24.5 ± 2.7 (4)
RER	0.83 ± 0.02 (4)	0.84 ± 0.02 (4)	0.73 ± 0.01 (4)	0.73 ± 0.01 (4)
Serum parameters				
HbA1c (%)	4.4 ± 0.1 (10)	4.6 ± 0.1 (7)	4.6 ± 0.1 (7)	4.6 ± 0.0 (7)

All results are expressed as means ± SEM. Numbers of animals are given in brackets.

Body composition reflected body weight development. Under chow conditions, h-vaspinTG had a significantly higher percentage of fat mass (19.6 ± 0.8% vs. 13.3 ± 1.2%), though differences in individual AT-depot masses were not significant (**Figures 1G, H**). On the contrary, HFD-fed h-vaspinTG had significantly less body fat (42.4 ± 1.6% vs. 36.1 ± 1.3%) and absolute as well as relative AT fat pad masses were significantly lower (**Figures 1G-I**). Also, liver weights were significantly lower in h-vaspinTG, while BAT weights were not different between genotypes, irrespective of diet conditions (**Figures 1H, I**).

We next assessed energy intake and expenditure as well as locomotor activity of h-vaspinTG in metabolic chambers. Food intake was not different and both genotypes consumed less energy dense HFD food compared to chow (**Table 1**). There were no differences in respiratory exchange rate, which clearly reflected the switch to lipid substrate metabolism under HFD in both genotypes (**Table 1**). Under chow diet, h-vaspinTG exhibited a trend for lower oxygen consumption (VO_2_, **Figure 1J**) and energy expenditure (EE, **Figure 1K**). In control mice, HFD-feeding significantly suppressed VO_2_ and EE compared to chow, as expected. This effect did not occur in h-vaspinTG mice. VO_2_ and EE during the light and dark phase were significantly higher in HFD-fed h-vaspinTG mice compared to HFD-fed controls (**Figures 1J, K**). Body temperatures were not increased in h-vaspinTG (**Table 1**). Finally, there were no differences in locomotor activity (**Figures 1L, M**; movement in cage (XY), rearing (Z) and running wheel) between the genotypes, and both groups of mice showed reduced activity under HFD.

### Vaspin transgenic mice exhibit less adipocyte hypertrophy and ectopic lipid deposition in liver

**Figure 2:**
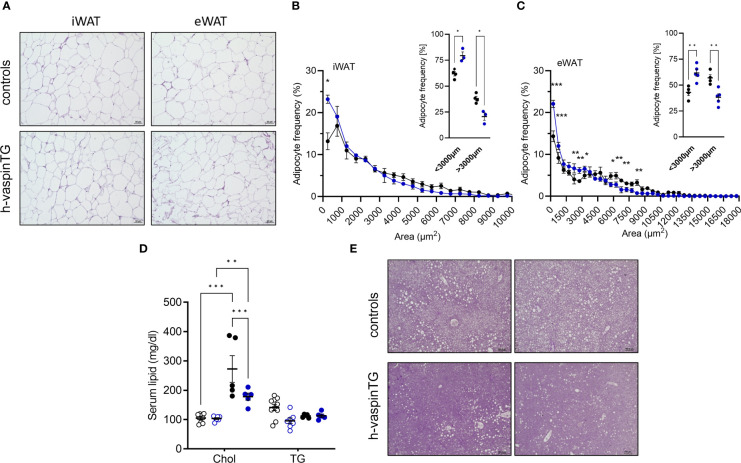
h-vaspinTG mice exhibit less hypertrophy in white adipose tissue (AT) and reduced fatty liver under high fat diet (HFD). **(A)** Representative histological H&E stained sections of inguinal (iWAT) and epididymal (eWAT) adipose tissue of HFD fed h-vaspinTG and control mice. Scale bar: 50 µm. **(B, C)** Adipocyte size distribution in iWAT **(B)** and eWAT **(C)** sections (n = 3-5 per group). **(D)** Cholesterol (Chol) and serum triglycerides (TG) (mg/dl) were measured in chow and HFD-fed h-vaspinTG and control mice. **(E)** Two representative histological PAS stained liver sections of HFD-fed h-vaspinTG and control mice. Scale bar: 100 µm. **(F)** Liver glycogen levels (µg) of chow and HFD-fed h-vaspinTG and control mice. (n = 5-10 per group). Data are presented as mean ± SEM. Statistical significance was calculated by multiple t-tests **(B, C)** or two-way ANOVA followed by Tukey’s *post-hoc* test **(D)** (*p ≤ 0.05, ** p ≤ 0.01, ***p ≤ 0.001).

AT histology revealed that the reduced fat mass in white AT depots of HFD-fed h-vaspinTG mice also featured smaller adipocytes, especially in eWAT (**Figures 2A-C**). As we also observed significantly lower liver weights in h-vaspinTG mice (**Figure 1H**), we assessed circulating lipids and ectopic lipid accumulation in livers of h-vaspinTG and control mice. Serum triglycerides were similar in h-vaspinTG and controls, but total circulating cholesterol, while increased after HFD in both genotypes, was significantly lower in h-vaspinTG mice (**Figure 2D**). Furthermore, liver histology revealed substantial fatty liver and ectopic lipid accumulation in in HFD-fed control mice (**Figure 2E**), which was clearly reduced in vaspin TG mice. In obese mice of both genotypes, glycogen levels were not different (**Figure 2E**, PAS stain).

### Vaspin transgenic mice show improved metabolic profile after HFD

**Figure 3:**
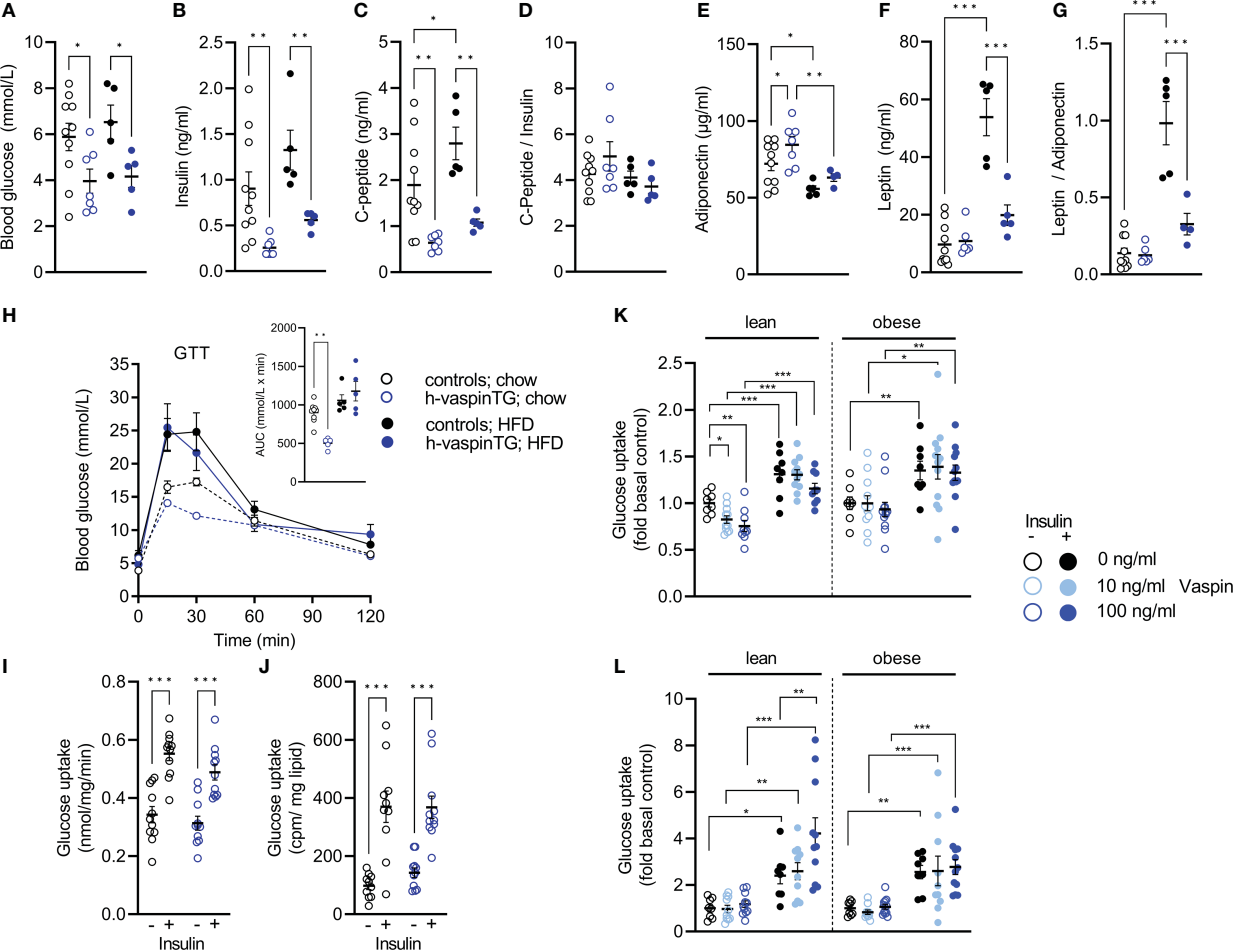
h-vaspinTG mice display healthier serum profile under high fat diet (HFD). **(A)** Blood glucose (mmol/L), **(B)** insulin (ng/ml) and **(C)** C-peptide levels (ng/ml), as well as **(D)** C-peptide/insulin ratio, **(E)** adiponectin (µg/ml) and **(F)** leptin levels (ng/ml), as well as **(G)** adiponectin/leptin ratio of h-vaspinTG and control mice fed a chow or HFD for 23 weeks. **(H)** Glucose tolerance tests (GTT) and area under the curve (AUC, presented in insert) in h-vaspinTG and control mice fed a chow or HFD. **(I, J)** *Ex vivo* glucose uptake in **(I)** EDL muscle (mmol/mg/min) and **(J)** epididymal adipocytes (cpm/mg lipid) of h-vaspinTG and control mice fed a chow or HFD (n = 5-10). **(K, L)** Relative *ex vivo* glucose uptake in **(K)** EDL muscle and **(L)** epididymal adipocytes from lean and obese C57BL/6N mice, treated with either 10 ng/ml or 100 ng/ml recombinant vaspin and compared to untreated controls (n = 8-12). Data are presented as mean ± SEM. Statistical significance was calculated by one-way ANOVA followed by Fisher’s LSD test (*p ≤ 0.05, ** p ≤ 0.01, ***p ≤ 0.001).

Both, under chow- and HFD-fed conditions, h-vaspinTG mice had significantly lower fasting blood glucose levels (**Figure 3A**). Also, free insulin levels were significantly lower (by ~70%) in h-vaspinTG mice compared to controls. Furthermore, while in control mice insulin levels increased by ~50% (p=0.08) under HFD, insulin levels in h-vaspinTG doubled, but levels were still below chow-fed controls and the increase did not reach statistical significance (**Figure 3B**). This was clearly due to reduced insulin secretion, as C-peptide levels were mirroring insulin levels in h-vaspinTG (**Figure 3C**), resulting in similar C-peptide/insulin ratios in both genotypes, irrespective of diet (**Figure 3D**). HbA1c was not different under all conditions (**Table 1**). Circulating levels of adiponectin, a marker of insulin sensitivity and metabolic health, were significantly higher in h-vaspinTG under chow diet, but were significantly decreased in both genotypes in response to the HFD (**Figure 3E**). Leptin on the other hand, was not different in chow-fed mice of both genotypes, and highly increased (~5-fold) in HFD-fed controls (**Figure 3F**). Reflecting the significantly reduced fat mass in h-vaspinTG after HFD, leptin levels in these mice were significantly lower compared to controls (by ~60%), albeit higher than in chow-fed controls (~2-fold) (**Figure 3F**). This resulted in a much healthier leptin/adiponectin ratio, which was highly increased in controls after HFD, but not significantly different in h-vaspinTG, when compared to chow-fed controls (**Figure 3G**). The beneficial serum profile of h-vaspinTG did not translate into improved glucose tolerance, and both genotypes showed obesity-induced impaired glucose clearance in the GTT (**Figure 3H**). Yet, h-vaspinTG mice had improved glucose tolerance under chow-fed conditions (**Figure 3H**). To assess insulin sensitivity more directly in muscle and AT, we performed ex vivo glucose uptake in isolated muscle and epididymal white AT. There were no differences in glucose uptake in both tissues (**Figures 3I, J**). As effects of vaspin on human myotubes have been reported before (18), we also assessed effects of recombinant vaspin on insulin-stimulated glucose uptake in EDL muscle and epididymal adipocytes of lean and obese controls *ex vivo* (**Figures 3K, L**). In muscle, vaspin had no effects on insulin-stimulated glucose uptake independent of obesity, but reduced basal uptake in muscle of lean mice. In eWAT adipocytes, vaspin did dose dependently increase insulin-stimulated glucose uptake, but only in lean mice.

These data indicate an improved metabolic profile based on serum markers, but did not translate into improved glucose metabolism and responsiveness to insulin in h-vaspinTG mice.

## Discussion

Vaspin exerts multiple beneficial effects in the context of obesity and especially obesity-related comorbidities, as well as liver and vascular inflammation (reviewed in (11, 12)). Important tools to study and establish the function of this intriguing serpin were vaspin-knockout and vaspin transgenic mouse models (10, 19, 20).

Similar to the previously described vaspinTG mouse (m-vaspinTG) (10), the new mouse line reported here features AT specific overexpression using the aP2 promotor. In contrast, the new line expresses the human *SERPINA12* transgene, while the initial model overexpressed the mouse *serpinA12* gene. It is important to note that key residues regulating vaspin function and specificity identified studying human vaspin (21, 22) are all conserved in the mouse ortholog. Among these are the reactive center loop with the protease cleavage site, regulatory exosites as well as the heparin binding site. Also, both ortholog proteins are glycoproteins, but differ in some glycosylation sites (23). They share one glycosylation site and while human vaspin exhibits two more glycosylation sites located on the top of the serpin molecule, mouse vaspin features only one additional glycosylation site located on the back of the molecule. Protease inhibition, heparin binding and thermal stability are comparable for both orthologs (23).

Vaspin expression levels are significantly higher in the new h-vaspinTG model. While initial m-vaspinTG mice had a 2-3xfold increased circulating vaspin levels, circulating vaspin levels in the h-vaspinTG mouse are three orders of magnitudes higher than in controls. Vaspin serum levels in humans are typically in the range of 0.5 - 1.5 ng/ml (6) and subpopulations have been reported with vaspin levels up to >30 ng/ml (24). Furthermore, there is significant diurnal variation in vaspin serum levels with the nocturnal peak reaching 250% of the daily minimum (25). In addition, vaspin interactions with cell surface receptors (10) or proteoglycans (21) will have significant effects on local concentrations of vaspin at or near the cell surface, that may be very different from the circulating levels. This is also reflected in concentrations of recombinant vaspin used in the literature to study cellular or tissue specific effects of vaspin (ranging from 1ng/ml up to 1µg/ml, with most of the studies investigating effects of vaspin in in the 100-500 ng/ml range). Thus, the h-vaspinTG mouse model clearly exhibits supraphysiologic levels of vaspin, but considering the variation observed in the human populations and also with respect to the *in vitro* data, these do not represent such extreme levels, which may result in effects that may not be physiologically relevant.

Together, the phenotype of the new h-vaspinTG mouse recapitulates and confirms the improved metabolic phenotype of the m-vaspinTG mouse, in part with larger effect size. Yet, there are also interesting differences in the high level-vaspin expressing h-vaspinTG mouse. First of all, both vaspinTG mouse lines are at least partly protected from HFD-induced obesity, but the final differences in weight gain after 20+ weeks on the HFD were much more pronounced in h-vaspinTG mice. On average, h-vaspinTG mice had gained ~15% less body weight after the HFD compared to controls (vs. ~8% in m-vaspinTG). This was mainly due to a significant reduction in less total body fat (~6%), and here especially of the iWAT depot. Both vaspin transgenic lines showed reduced hypertrophy under HFD-conditions. In the m-vaspinTG mouse, no direct explanation was found for reduced weight gain under HFD. There were no obvious differences in energy intake, energy expenditure (EE) and activity compared to controls, only a slightly increased RER may indicate alterations in metabolic substrate utilization in m-vaspinTG (10). This is different in the new model, where we did observe significant differences in VO_2_ and EE, albeit no changes in activity and RER. HFD-fed h-vaspinTG exhibited higher EE, both during the light and dark phase compared to HFD-fed controls. Here, the reduction in EE observed in mice fed a HFD (26), was not apparent in h-vaspinTG mice, despite both genotypes showing the expected reduction in activity under HFD. In HFD-fed h-vaspinTG mice, 24h EE was almost exactly the same as in chow-fed h-vaspinTG mice, with an increase during the light phase offsetting a slight reduction during the night phase. This increase in EE may be a key mechanism limiting weight-gain under HFD, as we did not observe differences in activity and food intake. While we and others have shown that acute vaspin application (intraperitoneally and intracerebroventricular) reduced food intake in obese and insulin resistant *db/db* mice (27–29), this effect seems to be lost, desensitized or compensated under conditions of chronically increased vaspin levels. Interestingly, under chow conditions the observation was the contrary, with h-vaspinTG mice showing a trend for lower VO_2_ and EE both during the light and dark phase, in line with a tendency for higher body weights at the end of the study. The significant induction of vaspin expression in activated BAT may link vaspin and thermogenesis (30), but how vaspin may regulate whole-body EE will be the focus of future investigations.

Reduced responsiveness to the HFD in h-vaspinTG mice was accompanied by a 60% reduction in circulating leptin and significant improvements in fasting blood glucose and insulin levels, as well as lower cholesterol levels at the end of the study. Notably, m-vaspinTG mice also exhibited an improved profile of circulating markers, halfway through the study (10 weeks under the HFD), however most of the parameters reached levels of control mice at the end of the study (10). This may in part be explained by the less pronounced difference in body weight in the HFD-fed m-vaspinTG compared to controls. Yet, m-vaspinTG mice exhibited significantly improved glucose and insulin tolerance *in vivo* after HFD compared to controls. Strikingly, we did not observe this in the h-vaspinTG model, and also *ex vivo* glucose uptake in muscle and AT of HFD-fed h-vaspinTG mice was not different. Peripheral vaspin effects on insulin sensitivity are controversially discussed. Application of recombinant vaspin increases the effect of an acute insulin bolus on blood glucose levels in insulin tolerance tests (7, 8). Clamp studies in *db/db* mice did not show increased glucose uptake in hyperinsulinemic-euglycemic clamps after acute vaspin administration (8), but chronic treatment (1µg/kg for 8 weeks) increased glucose uptake in rats (9). For both studies, resulting circulating h-vaspin levels post treatment were not reported. Clamp data from both the m-vaspinTG and h-vaspinTG mice are not available, but we did not observe improvement of insulin-mediated glucose uptake in muscle and AT of h-vaspinTG *ex vivo*. In human myotubes from a donor with obesity, vaspin had a significant effect on insulin-stimulated glucose uptake (18). We could not confirm this finding in *ex vivo* glucose uptake in muscle from lean and obese control mice. We did find a beneficial effect on insulin-stimulated glucose uptake in eWAT samples from lean mice incubated with recombinant vaspin, in line with our findings in vaspin-overexpressing 3T3-L1 adipocytes (31). In the liver, the h-vaspinTG mice clearly confirmed the protective effect of vaspin expression from HFD-induced fatty liver, with a significant reduction in ectopic lipid deposition compared to obese controls.

It has to be noted, that in addition to the higher circulating vaspin levels in the h-vaspinTG compared to the m-vaspinTG mice, phenotypic differences between these vaspin transgenic lines related metabolism and EE are likely affected by different genetic background, diet composition and genotype-diet interactions (32–34).

In conclusion, we report a novel human vaspin-transgenic mouse line, which recapitulates many previous findings using a low-level expressing m-vaspinTG mouse line, and in addition shows an increase in EE under HFD-conditions explaining the body weight phenotype. This new mouse model will be a valuable tool to delineate whole-body and tissue- or cell-specific effects of vaspin, not only in the context of obesity.

## Data availability statement

The original contributions presented in the study are included in the article/supplementary material. Further inquiries can be directed to the corresponding authors.

## Ethics statement

The animal study was reviewed and approved by Landesdirektion Leipzig, Sachsen, Germany, TVV26-16, State Ministry of Agriculture, Nutrition and Forestry, State of North Rhine-Westphalia, Germany, Reference number 84-02.04.2016.A430, Ethics Committee in Animal and Human Experimentation of the Universitat Autònoma de Barcelona, Spain.

## Author contributions

JB and PK conceived, designed, and supervised the study. JB, IE, AP, and FB generated vaspin transgenic mice. IR, JW, SK, JH, and JB conducted experiments and analyzed data. RB contributed serum measurements. AC and HA-H contributed ex vivo glucose uptake data. NK helped with animal experiments. IR and JH wrote the manuscript with input from PK and JB. All authors contributed to the article and approved the submitted version.
